# Intravenous lidocaine reduces pain intensity and attenuates the increase in plasma norepinephrine levels after hysterectomy

**DOI:** 10.5339/qmj.2025.81

**Published:** 2025-09-10

**Authors:** Andi Muhammad Takdir Musba, Srimulyanto Sardi, Ratnawati

**Affiliations:** 1 Universitas Hasanuddin Fakultas Kedokteran, Makassar, Indonesia *Email: takdir1974@gmail.com

**Keywords:** Intravenous lidocaine, spinal anesthesia, laparotomy, hysterectomy, analgesic

## Abstract

**Background:**

Intravenous lidocaine is used to treat acute postoperative pain by reducing discomfort, accelerating mobilization, and shortening hospitalization. This study evaluated the effects of intravenous bolus and continuous lidocaine infusion on pain intensity, fentanyl requirements, and plasma norepinephrine levels in hysterectomy surgery under spinal anesthesia.

**Methods:**

A double-blinded randomized clinical trial was conducted on 46 patients undergoing hysterectomy with spinal anesthesia. The lidocaine group (*n* = 23) received a 2% lidocaine bolus (1.5 mg/kg) followed by a continuous infusion (1 mg/kg/hour) for 24 hours. The control group (*n* = 23) received 0.9% NaCl. Resting and moving numeric rating scale (NRS) pain scores, total fentanyl requirements, and norepinephrine levels were recorded at 4, 6, 12, and 24 hours postoperatively.

**Results:**

Statistically significant differences in resting pain NRS were observed at the 4th, 6th, 12th, and 24th hours (0.52 ± 0.51 vs. 1.52 ± 0.51, 0.61 ± 0.58 vs. 1.52 ± 0.51, 0.52 ± 0.51 vs. 1.74 ± 0.81, and 0.52 ± 0.51 vs. 1.52 ± 0.51, respectively; *p* < 0.001). The reduction in pain score on the moving NRS in the lidocaine group was significantly greater at 6 hours (2.22 ± 0.79 vs. 2.91 ± 1.04; *p* = 0.001) and 12 hours (2.26 ± 0.75 vs. 2.57 ± 1.12; *p* < 0.001) postoperatively compared to the control group. Total fentanyl requirements were significantly lower in the lidocaine group (103.04 ± 33.63 mcg vs. 421.74 ± 74.32 mcg; *p* < 0.001). Plasma norepinephrine changes differed significantly at T6–T0 (−0.66 ± 1.81 pg/mL vs. 1.98 ± 1.69 pg/mL; *p* < 0.001) and T24–T0 (−1.74 ± 1.94 pg/mL vs. 1.53 ± 1.12 pg/mL; *p* < 0.001). No side effects were observed.

**Conclusion:**

Intravenous bolus and continuous lidocaine infusion reduced postoperative pain intensity, fentanyl requirements, and norepinephrine levels without significant side effects.

## 1. INTRODUCTION

Postoperative pain mostly consists of acute nociceptive pain due to tissue injury, which is characterized by peripheral and central sensitization of the nervous system. During surgery, damage to body tissue is followed by an inflammatory response, resulting in the emergence of noxious stimuli that cause sensitization of the sensory nervous system, characterized by hyperalgesia, allodynia, and prolonged pain.^1,2^

Lidocaine exerts analgesia, anti-hyperalgesia, and anti-inflammatory effects. This effect is influenced by multiple mechanisms, including sodium channels, G protein–coupled receptors, and N-methyl-D-aspartate (NMDA) receptors blockade. Eventually, sodium channel blockade occurs and results in inhibition of spontaneous neuronal activity, thereby reducing neuronal hyperactivity and increasing postoperative analgesia. Lidocaine has a greater effect when given perioperatively, where nociceptive input has not occurred, and may weaken the stress response by suppressing sympathetic reflex inhibition during surgery.^3,4^ For analgesia, the intravenous lidocaine dose typically includes an initial bolus of 1 to 2 mg/kg and a continuous infusion of 0.5 to 3 mg/kg/hour, with 1 to 2 mg/kg/hour being the most commonly reported and effective range.^3–5^

Major components in pain perception and analgesic requirement consisted of endogenous opioids (enkephalins, β-endorphins, dynorphins) and catecholamines (epinephrine, norepinephrine, dopamine).^6^ Norepinephrine is a neurotransmitter in the central nervous system that is stored in vesicles and released via external Ca^2+^ concentration-dependent exocytosis. Norepinephrine can also be released under resting conditions into the extraneuronal space, resulting in high levels of catecholamine with a lasting tonic effect on non-synaptic receptors.^7^ Postoperative pain causes a significant sympathetic stress response, where norepinephrine levels increase as the pain increases.^8^

Hysterectomy is a surgical procedure that involves the removal of the uterus through an incision in the abdomen, commonly performed for conditions such as uterine fibroids, abnormal uterine bleeding, and pelvic organ prolapse. It remains one of the most frequently conducted surgeries for women, with approximatively 21.1% of women in the United States aged 18 years or older having undergone a hysterectomy as of 2021.^9^ While minimally invasive techniques like laparoscopic and vaginal hysterectomy are increasingly preferred, laparotomy is still a viable option for certain clinical scenarios, particularly when extensive surgical intervention is required. Pain management during and after this procedure typically includes general or regional anesthesia, along with postoperative strategies such as epidural analgesia and intravenous medications.^10^

Laparotomy is associated with significant postoperative pain, making it an ideal candidate for evaluating alternative pain management strategies. Given the high volume of these surgeries performed annually, the findings could have substantial implications for improving pain management protocols.^11^

This study sought to evaluate the effect of an intravenous bolus of lidocaine 1.5 mg/kg followed by continuous infusion of 1 mg/kg/hour for 24 hours after laparotomy hysterectomy surgery under spinal anesthesia on pain intensity, total fentanyl requirement, and plasma norepinephrine level.

## METHODS

### 2.1 Study Design

This study was a randomized, double-blind clinical trial conducted between February and June 2022 at Dr. Wahidin Sudirohusodo Hospital, a public hospital in Makassar, Indonesia, and its associated hospital.

### 2.2 Population and Study Sample

Patients scheduled for elective hysterectomy surgery under the laparotomy technique with spinal anesthesia were invited to participate in the study. The inclusion criteria used were American Society of Anesthesiologists (ASA) physical status of I–II,^12^ age of 30 to 65 years, and body mass index (BMI) of 18.5 to 30 kg/cm^2^. Exclusion criteria included contraindications to spinal anesthesia, a history of allergies to the study medications, liver or kidney failure, cardiovascular disease (including arrhythmias and atrioventricular block), or refusal to participate. Drop-out criteria included unsuccessful spinal anesthesia, conversion to general anesthesia, surgical duration exceeding 2 hours and 30 minutes, bradycardia, or participant withdrawal from the study. The required sample size was calculated using power analysis based on the mean difference in pain numeric rating scale (NRS) scores with a significance level (α) of 0.05 and power (1 − β) of 80A. The analysis indicated that a minimum of 20 participants per group was needed, and a total of 46 participants were enrolled to account for potential dropouts.

### 2.3 Study Procedure

Participants were randomly allocated to either the lidocaine group (Group A) or the control group (Group B) using a computer-generated randomization sequence. Group A received an intravenous bolus of 2% lidocaine (1.5 mg/kg) followed by a continuous infusion of 1 mg/kg/hour via syringe pump for 24 hours postoperatively, while Group B received a placebo (0.9% NaCl) administered in the same manner and dosage as the lidocaine. Before the surgery, the subjects were fasted for 6 hours. Blood samples for plasma norepinephrine level were taken 2 hours before the surgery (T0). Spinal anesthesia was performed using 15 mg of 0.5% hyperbaric bupivacaine with 25 mcg of fentanyl as an adjuvant. Surgery was initiated after the sensory block reached the level of thoracic vertebrae VI with a Bromage scale of 3/3. Blood samples were taken 1 hour after the surgical incision (T1). Group A received a bolus of 2% lidocaine 1.5 mg/kg intravenously followed by a continuous infusion of 1 mg/kg/hour via syringe pump for 24 hours postoperatively, while group B received NaCl 0.9% at the same dose as group A. Blood samples were taken 6 hours (T6) and 24 hours (T24) after surgery. Pain intensity was measured using the NRS while resting and when moving (coughing, turning right and left, or sitting) 4, 6, 12, and 24 hours after surgery. The lidocaine infusion was initiated immediately after the end of surgery as per protocol and was not dependent on a threshold NRS score or patient-reported pain intensity. Patient-controlled analgesia device was used to document total fentanyl requirements for 24 hours postoperatively, with a demand dose of 20 mcg and a 10-minute lock-out interval without background infusion. The subjects received paracetamol 1000 mg/6 hours and ibuprofen 400 mg/6 hours orally as part of multimodal analgesia. Side effects such as nausea/vomiting, allergy, hypotension, bradycardia, sedation, and local anesthetic systemic toxicity (LAST) were noted and recorded.

In our hospital, postoperative pain management and monitoring are supported by a multidisciplinary Pain Team comprising anesthesiologists and trained nurses. This team conducts routine assessments to evaluate analgesic efficacy and detect adverse effects such as respiratory depression, sedation, hypotension, bradycardia, nausea, and signs of LAST. Monitoring is particularly emphasized during the first 24 hours postoperatively, coinciding with the period of lidocaine infusion and peak opioid use.

### 2.4 Data Analysis

SPSS 25.0 (IBM^®^) was used to analyze the data. Data normality was tested with the Shapiro-Wilk. The comparison of mean pain NRS values between the groups was analyzed using a paired t-test for normally distributed data or the Wilcoxon test for non-normally distributed data. Further statistical tests were conducted to analyze the mean differences in total opioid requirements and changes in plasma norepinephrine levels between the groups. An unpaired t-test was used for normally distributed data, while the Mann-Whitney test was applied for non-normally distributed data. A difference was considered statistically significant if the *p*-value was less than 0.05.

### 2.5 Ethical Approval

The study was approved by the Institutional Review Board of Dr. Wahidin Sudirohusodo Hospital, Makassar, Indonesia, and Hasanuddin University Faculty of Medicine (approval reference number: 8/UN4.6.4.5.31/PP36/2022, protocol number: UH21120772). Written informed consent was obtained from all participants before enrollment in the study. The consent process included detailed information about the study’s purpose, procedures, potential risks, and benefits. Participants were informed of their right to withdraw from the study at any time without any consequences to their medical care. The study was conducted in accordance with the Declaration of Helsinki and relevant ethical guidelines.

## 3. RESULTS

### 3.1 Sample Characteristics

There were 46 participants in the study (23 participants in each group). There were no significant differences in age (*p* = 0.349), weight (*p* = 0.584), BMI (*p* = 0.127), length of surgery (*p* = 0.526), and blood loss (*p* = 0.414) between the groups (Table 1).

### 3.2 Pain Intensity

At 4, 6, 12, and 24 hours postoperatively, pain NRS scores were used to compare the groups’ pain intensity (Table 2). There were statistically significant differences in changes of resting pain NRS at the 4th, 6th, 12th, and 24th hour measurements (0.52 ± 0.51 vs. 1.52 ± 0.51, 0.61 ± 0.58 vs. 1.52 ± 0.51, 0.52 ± 0.51 vs. 1.74 ± 0.81, and 0.52 ± 0.051 vs. 1.52 ± 0.51, respectively; *p* < 0.001) between groups, with Group A showing significantly lower pain intensity compared to Group B. Furthermore, the pain intensity for moving pain NRS at the 6th and 12th hours demonstrated a statistically significant difference (2.22 ± 0.79 vs. 2.91 ± 1.04 and 2.26 ± 0.75 vs. 2.57 ± 1.12, respectively; *p* < 0.001) between the groups, with Group A showing significantly lower pain intensity compared to Group B.

### 3.3 Fentanyl Requirement

Fentanyl total requirement over 24 hours was tested by the independent t-test. The mean 24-hour use of fentanyl in Group A (103.04 mcg) was significantly lower than that of Group B (421.74 mcg; *p* < 0.001; Table 3).

### 3.4 Plasma Norepinephrine Level and Side Effects

In T1–T0, there was an increase in plasma norepinephrine level in both groups. In T6–T0 and T24–T0, there was a decrease in plasma norepinephrine level in Group A, and an increase in Group B. There was a significant difference in changes of plasma norepinephrine level in T1–T0, T6–T0, and T24–T0 between Group A and Group B (*p* < 0.05; Table 4). No side effects such as nausea/vomiting, allergy, hypotension, bradycardia, sedation, and LAST were reported in both groups (Table 5).

## 4. DISCUSSION

Postoperative pain, which the patient experiences as a painful sensory and emotional event, is a complex physiological response to tissue injury, visceral distention, and disease response. Postoperative sensitization will result in suffering for the patient, which may increase postoperative morbidity and mortality. Therefore, efforts to control postoperative pain must focus on both preventing and minimizing the development of the sensitization process.^2^ In addition to the type of surgery and administration of analgesics, postoperative pain can also be affected by age, gender, preoperative pain, anxiety, and incision size.^13^ These factors were used in the exclusion criteria to prevent confounding factors in this study. Some of these factors were analyzed using demographic statistics and showed no significant results. Data analysis on sample characteristics such as age, weight, BMI, duration of surgery, and amount of blood loss showed homogeneous results (Table 1); hence, the sample can be used in research.

### 4.1 Pain Intensity

The active metabolite of lidocaine acts by blocking sodium channels in the peripheral and central nerves, thus suppressing ectopic impulses to afferent nerve cells and inhibiting dorsal horn polysynaptic reflexes in the spinal cord, which ultimately inhibits the delivery of pain impulses without conduction blockade and reduces postsynaptic receptor depolarization due to NMDA receptors.^14^

Resting pain NRS score in the lidocaine group was significantly decreased at all measurement times compared to the control group. The same result was found on the moving pain NRS score, but only at 6 and 12 hours postoperatively. This result can be explained by the pharmacology of spinal anesthesia that there may still be an effect from spinal anesthesia at 4 hours postoperatively, while the pain at 24 hours postoperatively has been reduced by the effect of lidocaine, which maintains plasma levels for 24 hours, where the noxious stimulus becomes lower. Kaba et al and Baral et al reported similar results, where an intravenous bolus of 2% lidocaine 1.5 mg/kg followed by continuous infusion 1.5 to 2 mg/kg/hour resulted in lower levels of pain and requirement for opioids in the lidocaine group.^15,16^

### 4.2 Total Fentanyl Requirement

Perioperative pain is usually managed with opioid analgesics. However, opioids could have negative side effects such as respiratory depression, nausea, vomiting, and drowsiness.^17^ Additionally, opioid use–related acute tolerance may lead to an increase in postoperative pain. Therefore, a multimodal analgesic regimen is needed to reduce perioperative opioid requirements.^18^ The lidocaine group in this study required less total fentanyl over 24 hours. Previous study by Kaba et al, which used higher doses of lidocaine, demonstrated a reduced opioid requirement in the lidocaine group compared to placebo (8 [5–18] vs. 22 [14–36] mg; *p* = 0.005).^15^

Recent research has demonstrated that intravenous lidocaine administration during hysterectomy significantly reduces postoperative opioid consumption. For instance, a randomized controlled trial by Shady et al found that adjunctive intravenous lidocaine infusion in overweight and obese women undergoing abdominal hysterectomy led to lower pain scores and decreased pethidine requirements compared to placebo.^19^ A meta-analysis by Tang et al included nine randomized controlled trials, five of which involved abdominal hysterectomy. The study found that intravenous lidocaine significantly reduced postoperative pain at 2, 6, 8, and 24 hours, as well as total morphine consumption within the first 24 hours. Furthermore, lidocaine reduced the incidence of postoperative nausea and vomiting, highlighting its opioid-sparing and recovery-enhancing effects specifically in hysterectomy patients.^20^ Conversely, Bryson et al, in a randomized controlled trial involving 90 women undergoing abdominal hysterectomy, found no significant difference in opioid consumption, pain scores, or length of hospital stay between the lidocaine and placebo groups. However, their study limited lidocaine administration to the intraoperative period only. This difference in duration of infusion may partially explain the inconsistent outcomes.^21^ The accumulating evidence suggests that extended lidocaine administration, beyond the intraoperative period, may be more effective in optimizing postoperative recovery in abdominal hysterectomy.

Administration of continuous lidocaine in several non-abdominal surgeries also showed the same results. Sipahutar et al used intravenous bolus of lidocaine 2% 1.5 mg/kg followed by continuous infusion 1 mg/kg in breast surgery patients, which resulted in reduced requirement for postoperative fentanyl rescue in the lidocaine group compared to placebo (*p* < 0.001; 0 [0–25] mcg vs. 50 [25–75] mcg).^22^ Farag et al administered continuous intravenous lidocaine at 2 mg/kg/hour for 8 hours postoperatively following complex spine surgery, demonstrating that the lidocaine group was noninferior in terms of mean morphine equivalent consumption (*p* = 0.011; 55 [36–84] and 74 [49–111] mg, respectively) compared to placebo.^23^ Analgesic requirements are directly proportional or reciprocally related to pain intensity, where more opioids will be required in moderate to severe pain than in mild pain.^6^ Lidocaine exhibited opioid sparring effect, and using larger doses of lidocaine can increase its efficacy, while closely monitoring the side effects.^3^

The total dose of analgesia administered not only impacts patient outcomes but also carries financial implications. A reduced requirement for analgesics, as observed in this study, can lower hospital lengths of stay and overall treatment costs. Additionally, minimizing analgesic use reduces the likelihood of associated side effects, leading to shorter recovery times and potentially decreasing hospital stays.^24^ Although postoperative pain management may not be as costly as other components of surgical care, it remains crucial due to its significant impact on patient outcomes and the overall efficiency of the healthcare system.^25^ These financial and clinical benefits further underscore the importance of optimizing pain management strategies to enhance both patient care and healthcare system efficiency.

The intravenous lidocaine regimen, comprising a bolus followed by continuous infusion, was selected specifically for this clinical trial based on evidence from previous studies demonstrating its potential analgesic and anti-inflammatory benefits. It is not part of the routine postoperative pain management protocol for hysterectomy at our hospital, which typically includes paracetamol and Nonsteroidal Anti-Inflammatory Drugs (NSAIDs), with opioids administered as needed for breakthrough pain.

### 4.3 Plasma Norepinephrine Level

Tissue injury in surgery can trigger an inflammatory reaction by releasing inflammatory agents such as histamine, bradykinin, and prostaglandins that cause sympathetic hyperactivity.^26^ In this study, there was a decrease in plasma norepinephrine levels after administration of lidocaine (Table 4). This result was consistent with those reported by Wallin et al, where urinary norepinephrine levels were significantly greater in the control group on the second postoperative day than in the lidocaine group during abdominal surgery.^27^ The decreased norepinephrine level could also be associated with reduced fentanyl requirements in the lidocaine group, which concurred with Uribe-Rivera et al that higher plasma norepinephrine levels were associated with increased postoperative opioid requirements compared to the control group (29.1 [0–45] mg vs. 12 [0–71.5] mg; *p* = 0.0553).^6^

### 4.4 Side Effects

Lidocaine toxicity occurs when the plasma concentration exceeds 5 µg/mL. Early side effects may include perioral paresthesia, confusion, audio–visual disturbances, dysgeusia, and agitation. If the plasma concentration exceeds 12 μg/mL, loss of consciousness, convulsions, arrhythmias, and cardiac arrest may occur.^28,29^ The intravenous lidocaine consensus statement reported that the infusion rate of lidocaine up to 3 mg/kg/hour only resulted in an average plasma concentration of 2.6 (± 0.6) μg/mL.^30^ Side effects of LAST were not seen in this study, which showed that continuous administration of lidocaine 1 mg/kg/hour until 24 hours postoperatively does not exceed a plasma concentration of 5 μg/mL.

### 4.5 Limitations

This study has several limitations. The sample size was relatively small, which may affect the generalizability of the findings. Additionally, the study focused on a specific surgical procedure, hysterectomy, which limits the applicability of the results to other types of surgeries. Furthermore, the research was conducted in a single center and its associated hospital, rather than multiple sites, which may reduce the external validity of the conclusions. Future studies with larger, more diverse populations and involving multiple centers are recommended to validate these findings and assess their broader applicability.

## 5. CONCLUSION

Administration of intravenous bolus and continuous 2% lidocaine reduces pain intensity, fentanyl requirements, and attenuates the increase in plasma norepinephrine levels in hysterectomy patients without marked side effects. Therefore, 2% lidocaine may serve as an effective component of multimodal analgesia in the perioperative period. In addition to its analgesic benefits, lidocaine may also contribute to reduced hospital length of stay and overall healthcare costs. Further studies are warranted to evaluate its efficacy and economic impact across other surgical populations.

## Figures and Tables

**Table 1.** Sample characteristics.

**Characteristics**	**Group**				***p* value**
	**A (Lidocaine 2%; *n* = 23)**		**B (NaCl 0.9%; *n* = 23)**		
	**Mean ± SD**	**Median** **(Min–Max)**	**Mean ± SD**	**Median** **(Min–Max)**	
Age (years)	46.78 ± 3.37	47 (40–53)	46.70 ± 4.51	46 (40–57)	0.349^a^
Weight (kg)	57.74 ± 5.59	57 (50–65)	58.57 ± 3.75	60 (50–65)	0.584^b^
BMI (m/kg^2^)	23.04 ± 1.93	22.9 (20–27.1)	22.70 ± 1.10	22.4 (20.5–25.4)	0.127^a^
Duration of surgery (minutes)	97.83 ± 13.46	97.83 (90–120)	100.43 ± 14.61	100.43 (90–120)	0.526^b^
Blood loss (mL)	347.83 ± 43.89	350 (280–420)	335.22 ± 54.00	340 (250–420)	0.414^b^

Data were analyzed with an unpaired t-test (a) and Mann-Whitney test (b).

**Table 2.** Comparison of changes in the numeric rating scale (NRS) between groups.

**NRS**	**Time**	**Group**				***p* value**
		**A (Lidocaine 2%; *n* = 23)**		**B (NaCl 0.9%; *n* = 23)**		
		**Mean ± SD**	**Median (Min–Max)**	**Mean ± SD**	**Median (Min–Max)**	
Resting	T4–T0	0.52 ± 0.51	1.00 (0–1)	1.52 ± 0.51	2.00 (1–2)	<0.001
	T6–T0	0.61 ± 0.58	1.00 (0–2)	1.52 ± 0.51	2.00 (1–2)	<0.001
	T12–T0	0.52 ± 0.51	1.00 (0–1)	1.74 ± 0.81	2.00 (1–4)	<0.001
	T24–T0	0.52 ± 0.051	1.00 (0–1)	1.52 ± 0.51	2.00 (1–2)	<0.001
Moving	T4–T0	2.22 ± 0.79	2.00 (1–3)	2.35 ± 0.93	2.00 (1–5)	0.164
	T6–T0	2.22 ± 0.79	2.00 (1–3)	2.91 ± 1.04	3.00 (2–6)	0.001
	T12–T0	2.26 ± 0.75	2.00 (1–3)	2.57 ± 1.12	2.00 (2–6)	<0.001
	T24–T0	1.22 ± 0.79	1.00 (0–3)	1.65 ± 0.71	2.00 (1–3)	0.161

Data were analyzed with the Mann-Whitney test. Values with *p* < 0.05 are considered statistically significant.

**Table 3.** Comparison of 24-hour fentanyl requirement between groups.

**Fentanyl**	**Group**				***p* value**
	**A (Lidocaine 2%)**		**B (NaCl 0.9%)**		
	***n***	**Mean ± SD**	***n***	**Mean ± SD**	
Total dose (mcg)	23	103.04 ± 33.63	23	421.74 ± 74.32	<0.001*

Data were analyzed with the independent/sample t-test. Values with *p* < 0.05 are considered statistically significant.

**Table 4.** Comparison of changes in plasma norepinephrine levels between groups.

**Cytokine**	**Time**	**Group**				***p* value**
		**A (Lidocaine 2%)**		**B (NaCl 0.9%)**		
		**Mean ± SD**	**Median** **(Min–Max)**	**Mean ± SD**	**Median** **(Min–Max)**	
Norepinephrine (pg/mL)	T1–T0	2.20 ± 2.29	1.30 (0.13–8.37)	3.42 ± 1.71	3.22 (0.49–7.44)	0.003
	T6–T0	−0.66 ± 1.81	−0.74 (−5.34 to 5.41)	1.98 ± 1.69	1.23 (0.06–5.93)	<0.001
	T24–T0	−1.74 ± 1.94	−1.03 (−6.57 to 0.30)	1.53 ± 1.12	1.17 (0.19–4.06)	<0.001

Data were analyzed with the Mann-Whitney test. Values with *p* < 0.05 are considered statistically significant.

**Table 5.** Comparison of side effects between groups.

**Variables**	**Group**	
	**A (Lidocaine 2%)**	**B (NaCl 0.9%)**
**Nausea/vomiting**		
No	23 (100.0%)	23 (100.0%)
Yes	0 (0.0%)	0 (0.0%)
**Allergy**		
No	23 (100.0%)	23 (100.0%)
Yes	0 (0.0%)	0 (0.0%)
**Hypotension**		
No	23 (100.0%)	23 (100.0%)
Yes	0 (0.0%)	0 (0.0%)
**Bradycardia**		
No	23 (100.0%)	23 (100.0%)
Yes	0 (0.0%)	0 (0.0%)
**Sedation**		
No	23 (100.0%)	23 (100.0%)
Yes	0 (0.0%)	0 (0.0%)
**Local anesthetic systemic toxicity (LAST)**		
No	23 (100.0%)	23 (100.0%)
Yes	0 (0.0%)	0 (0.0%)

Data were analyzed with Fisher’s Exact Test.

## References

[bib1] Pogatzki-Zahn EM, Segelcke D, Schug SA (2017). Postoperative pain-from mechanisms to treatment. Pain Rep.

[bib2] Elvidiansyah, Fuadi I, Sitanggang RH (2014). Comparison between the effect of single dose 150 mg and 300 mg pregabalin of Numeric Rating Scale value and post operative analgesia requirement in abdominal hysterectomy patients. J Anestesi Perioperatif.

[bib3] Herroeder S, Pecher S, Schönherr ME, Kaulitz G, Hahnenkamp K, Friess H (2007). Systemic lidocaine shortens length of hospital stay after colorectal surgery: a double-blinded, randomized, placebo-controlled trial. Ann Surg.

[bib4] Yon JH, Choi GJ, Kang H, Park JM, Yang HS (2014). Intraoperative systemic lidocaine for pre-emptive analgesics in subtotal gastrectomy: a prospective, randomized, double-blind, placebo-controlled study. Can J Surg.

[bib5] Groudine SB, Fisher HAG, Kaufman RP, Patel MK, Wilkins LJ, Mehta SA (1998). Intravenous lidocaine speeds the return of bowel function, decreases postoperative pain, and shortens hospital stay in patients undergoing radical retropubic prostatectomy. Anesth Analg.

[bib6] Uribe-Rivera A, Rasubala L, Machado-Perez AC, Ren YF, Malmström H, Carinci A (2021). Preliminary study of the impact of elevated circulating plasma levels of catecholamines on opioid requirements for acute surgical pain. J Clin Transl Sci.

[bib7] Sircuta C, Lazar A, Azamfirei L, Baranyi M, Vizi ES, Borbély Z (2016). Correlation between the increased release of catecholamines evoked by local anesthetics and their analgesic and adverse effects: Role of K(+) channel inhibition. Brain Res Bull.

[bib8] Ledowski T, Reimer M, Chavez V, Kapoor V, Wenk M (2012). Effects of acute postoperative pain on catecholamine plasma levels, hemodynamic parameters, and cardiac autonomic control. Pain.

[bib9] Harvey SV, Pfeiffer RM, Landy R, Wentzensen N, Clarke MA (2022). Trends and predictors of hysterectomy prevalence among women in the United States. Am J Obstet Gynecol.

[bib10] Gopalani SV, Dasari SR, Adam EE, Thompson TD, White MC, Saraiya M (2023). Variation in hysterectomy prevalence and trends among U.S. States and Territories-Behavioral Risk Factor Surveillance System, 2012–2020. Cancer Causes Control.

[bib11] Munisi I, Muhumba EK, Kibunto P, Shija R, Thomas R, Mgaya H (2024). Adequacy of postoperative pain management among patients undergoing laparotomy at Bugando Medical Centre. Mwanza, Tanzania. EAS J Med Surg.

[bib12] Daabiss M (2011). American Society of Anaesthesiologists physical status classification. Indian J Anaesth.

[bib13] Gan TJ (2017). Poorly controlled postoperative pain: prevalence, consequences, and prevention. J Pain Res.

[bib14] Rosow C, Dershwitz M, Longnecker D, Mackey S, Newman M, Sandberg W, Zapol W (2012). Pharmacology of opioid analgesic. Anesthesiology.

[bib15] Kaba A, Laurent SR, Detroz BJ, Sessler DI, Durieux ME, Lamy ML (2007). Intravenous lidocaine infusion facilitates acute rehabilitation after laparoscopic colectomy. Anesthesiology.

[bib16] Baral BK, Bhattarai BK, Rahman TR, Singh SN, Regmi R (2010). Perioperative intravenous lidocaine infusion on postoperative pain relief in patients undergoing upper abdominal surgery. Nepal Med Coll J.

[bib17] Cohen B, Ruth LJ, Preuss CV (2023). Opioid analgesics.

[bib18] Yon JH, Choi GJ, Kang H, Park JM, Yang HS (2014). Intraoperative systemic lidocaine for pre-emptive analgesics in subtotal gastrectomy: a prospective, randomized, double-blind, placebo-controlled study. Can J Surg.

[bib19] Shady NW, Farouk HA, Sallam HF (2021). Preemptive use of adjunctive IV lidocaine for enhanced recovery after abdominal hysterectomy for overweight and obese women: a prospective, randomized, double-blind, placebo-controlled study. Int J Reprod Contracept Obstet Gynecol.

[bib20] Tang P, Sun Q, Li Z, Tong X, Chen F (2025). Perioperative intravenous lidocaine infusion improves postoperative analgesia after hysterectomy: a systematic review and meta-analysis of randomized controlled trials. Int J Surg.

[bib21] Bryson GL, Charapov I, Krolczyk G, Taljaard M, Reid D (2010). Intravenous lidocaine does not reduce length of hospital stay following abdominal hysterectomy. Can J Anaesth.

[bib22] Sipahutar TC, Fuadi I, Bisri T (2013). The effect of intravenous lidocaine on Numeric Rating Scale value and postoperative fentanyl requirement in general anesthesia. J Anestesi Perioperatif.

[bib23] Farag E, Ghobrial M, Sessler DI, Dalton JE, Liu J, Lee JH (2013). Effect of perioperative intravenous lidocaine administration on pain, opioid consumption, and quality of life after complex spine surgery. Anesthesiology.

[bib24] Lee JS, Vu JV, Edelman AL, Gunaseelan V, Brummett CM, Englesbe MJ (2020). Health care spending and new persistent opioid use after surgery. Ann Surg.

[bib25] Bojic S, Ladjevic N, Palibrk I, Soldatovic I, Likic-Ladjevic I, Meissner W (2023). Cost-effectiveness of the perioperative pain management bundle a registry-based study. Front Public Health.

[bib26] Cao B, Xu Q, Shi Y, Zhao R, Li H, Zheng J (2024). Pathology of pain and its implications for therapeutic interventions. Signal Transduct Target Ther.

[bib27] Wallin G, Cassuto J, Hogstrom S, Linden I, Faxen A, Rimback G (1987). Effects of lidocaine infusion on the sympathetic response to abdominal surgery. Anesth Analg.

[bib28] Shalaby M, Sahni R, Hamilton R (2024). Local anesthetic systemic toxicity: awareness, recognition, and risk mitigation in the emergency department. Clin Exp Emerg Med.

[bib29] Abdullah S, Tokiran MF, Ahmad AA, Soh EZF, Makpol S, Sapuan J (2023). Safety of lidocaine during wide-awake local anesthesia no tourniquet for distal radius plating. J Hand Surg Glob Online.

[bib30] Foo I, Macfarlane AJR, Srivastava D, Bhaskar A, Barker H, Knaggs R (2021). The use of intravenous lidocaine for postoperative pain and recovery: international consensus statement on efficacy and safety. Anaesthesia.

